# Editorial: The role of healthcare delivery, payment and policy innovations in decreasing the global burden of chronic disease

**DOI:** 10.3389/fpubh.2022.972064

**Published:** 2022-09-14

**Authors:** Steven W. Howard, Rhonda BeLue, Joris van de Klundert

**Affiliations:** ^1^Health Services Administration, University of Alabama at Birmingham, Birmingham, AL, United States; ^2^Public Health, University of Texas at San Antonio, San Antonio, TX, United States; ^3^Business School, Adolfo Ibáñez University, Santiago, Chile

**Keywords:** chronic disease, healthcare innovation, health policy, value-base care, alternative payment

This Research Topic debuted in 2016 under the title *The Role of Financing, Delivery and Policy Innovations in Decreasing Chronic Disease Burdens*, and has been well received and widely read. In this new Research Topic, we aimed to reprise and refresh the topic of Innovations in Chronic Disease Care, with a renewed focus on value-based and other models of improved care delivery and payment.

The morbidity and mortality caused by chronic diseases increasingly dominates the global burden of disease. Prevention and treatment of these chronic conditions, such as diabetes, hypertension, heart disease, pulmonary conditions, requires the involvement of various formal provider organizations in primary care, specialists and hospitals, as well as of informal care givers. This Research Topic aimed to focus on improvement of health service networks, value-based payment arrangements, and health policies to prevent or more effectively treat chronic diseases. Such improvements often take the form of policy improvements (including financing, regulation, or otherwise), payment and insurance schemes (e.g., insurance, managed care, direct contracting, accountable care, value-based designs), and of interventions in service delivery (care coordination, care processes across organizations, information systems, communication, sharing of resources, etc). Often the most effective improvements involve multi-faceted interventions.

This Research Topic sought to advance the evidence base on effectiveness of interventions to improve the performance of networks servicing patients with chronic conditions, and value-based payment mechanisms aiming to improve outcomes and costs. The medical and health services literature often focuses on interventions addressing single provider organizations, episodes of the full chronic disease patient journey, and corresponding sub-processes. Less is published on more integrated approaches involving multiple components of the networks, especially research on approaches that include primary and/or secondary prevention. The prevention of onset, early-stage advancement, and subsequent complications and co-morbidities is necessary to manage the societal, organizational, and household-level costs of disease burden.

While addressing chronic disease management and prevention was historically the problem of middle and higher-income countries, improving access to chronic disease-related health care services and reducing the cost of care has become a global imperative. The growing complexity and burden of non-communicable disease, multiple morbidity, and the COVID-19 pandemic have increased demand for care. As a result, costs are increasing due to the rise of the global population, technological advances, and the increasing use of telehealth. When this happens concurrently with plunging GDP in an economic downturn, like in 2008–2009 and 2019–2021, the combined result can be devastating to national economies and public health (Figure 1, OECD, 2022).

**Figure 1:**
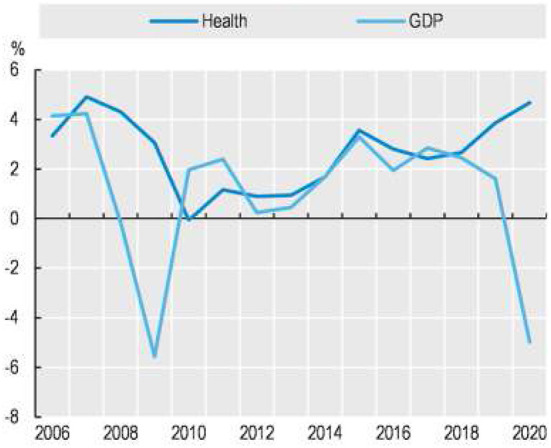
Annual real growth in per capita health expenditure and GDP, OECD, 2005–2020. https://www.oecd-ilibrary.org/sites/ae3016b9-en/1/3/7/1/index.html?itemId=/content/publication/ae3016b9-en&_csp_=ca413da5d44587bc56446341952c275e&itemIGO=oecd&itemContentType=book#figure-d1e10418.

In higher-income countries, including the OECD, spending as a percentage of GDP increased from 8.6 to 9.9% from 2010 to 2020 (OECD, 2022). While access in these countries is strong overall, costs require innovative strategies for containment. In many low and middle-income countries, healthcare spending and access lag the needs of the population.

This issue of Frontiers in Public Health features articles on alternative payment strategies and innovations in health care delivery by health services experts from around the world. Several themes converge across the featured articles including the role of reimbursement on health outcomes; the role of consumer knowledge and beliefs and healthcare cost and utilization; utilization patterns in government sponsored insurance programs and novel methods to improve the efficiency and reduce the cost of care for chronic conditions.

The articles selected cover a broad and diverse set of global efforts toward “value improvement” in health care. The original positioning of value-based healthcare in terms of improving outcomes that matter to patients over simply lowering cost is apparent across the studies (1). At the same time, the variety in outcomes and in the financial measures and instruments considered is notable, as is the variety in levels of intervention which range from national policy levels to (sub)organizational contexts. This diversity of policy and organizational context shows how the challenges for value improvement for multiple chronic conditions lie at various levels of the health system and differ across countries and conditions. Each of the studies provides evidence and insights with knowledge and inspiration for value improvement in other contexts. The effectiveness of improved insurance coverage and reimbursement are considered by Xie and Hu and by Ayubcha et al., who report improvements in self-reported health and reduced mortality, respectively. At the same time, Yan et al. provide evidence that such improvements may not be obtained equitably among all targeted patient populations, and more specifically find racial inequities for dementia patients. Reimbursement rates are obviously closely related to out-of-pocket spending, as analyzed in detail for Chronic Disease Syndrome by Close et al. Their analysis reveals the considerable costs involved for individual patients as well as for society at large. Together these financial analyses show the magnitude of the cost of chronic illnesses and how financial policy interventions can improve the outcomes that matter to patients.

Financial incentives might also be targeted to reduce cost and improve outcomes by prevention of illness. This perspective is taken by Salvado et al. who find that the effectiveness of financial incentives for professionals or organizations may vary depending on the type of prevention and incentive scheme. Ranjha et al. and Hosseinejad et al. also focus on efforts to improve the contributions made by professionals. For malaria prevention and elimination, and for community health services, respectively, they identify education and training of the workforce as an important driver of improvement, where Hosseinejad et al. identify a broader bundle of policy interventions needed to strengthen primary care by introducing community health nurses. Interestingly, on the patient side, Ahmad et al. show that education and knowledge may not suffice to produce the desired behaviors and not prevent unnecessary spending.

The studies of Howard et al. and Smeulders et al. focus primarily on outcomes for patients. Smeulders et al. explicitly distinguish outcomes of general validity and importance from outcomes whose validity is more limited. Such differences in validity of outcomes (and cost) that matter to patients echo recent explicit evidence that invalidate the general applicability of value-based healthcare measures (2). Thus, while the publications in the Research Topics strengthen the knowledge and evidence bases on *The Role of Financing, Delivery and Policy Innovations in Decreasing Chronic Disease Burdens*, they also remind us strongly that value improvement efforts need to be tailored toward the health systems, in particular the financial systems, and need to identify outcomes that matter to the patient in these contexts. The manuscripts published in this Research Topic showcase an inspiring collection of such value improvement research.

## Author contributions

All authors listed have made a substantial, direct, and intellectual contribution to the work and approved it for publication.

## Conflict of interest

The authors declare that the research was conducted in the absence of any commercial or financial relationships that could be construed as a potential conflict of interest.

## Publisher's note

All claims expressed in this article are solely those of the authors and do not necessarily represent those of their affiliated organizations, or those of the publisher, the editors and the reviewers. Any product that may be evaluated in this article, or claim that may be made by its manufacturer, is not guaranteed or endorsed by the publisher.
